# Progress in Surgery

**Published:** 1900-09-01

**Authors:** 


### Progress in Surgery.

### CEREBRAL SURGERY.

(*Continued from page 354.*)

**Cranial and Spinal Dermoids.**—F. Marsh[5] records, and gives illustrations of, a case in which a large dermoid on the forehead, simulating a meningocele, was successfully removed from a man of 20. The swelling, which had a circumference at its base of 11¼ inches, had been present since birth, but had increased considerably during the previous twelve months. There was no impulse or coughing, nor could the size of it be diminished by pressure. It was not translucent, but was fluctuant. The bone round the base was felt as a raised ridge. The contents of the cyst were hair, sebaceous matter, and epithelial *débris*. W. Hale White and A. D. Fripp communicated a paper to the Clinical Society in March[6] on a case of dermoid tumour growing within the spinal canal. The patient was a man of 26, who had begun to complain of loss of power in the legs in January, 1899. By the end of March there was complete paraplegia without muscular wasting, but with rigidity of the legs and excessive reflexes. The breathing was almost entirely diaphragmatic. All sensation was lost up to the nipples. Severe pain shooting down both intercosto-humeral nerves was complained of. There was incontinence of urine and cystitis. Marked tenderness over the second, third, and fourth dorsal spines was found. Caries and aneurysm were considered not to be present, the diagnosis being that there was some collection of fluid, or a new growth within the spinal canal irritating the second dorsal nerves, and compressing the cord below. The second, third, and fourth dorsal arches were removed, and a grey tumour situated on the outer surface of the dura-mater exposed, reaching out of sight above and below. Pieces of this were snipped away. The wound healed by first intention. The tumour proved on microscopical examination to be a dermoid cyst. Eight weeks later it was found that there was slight voluntary power over the legs, the reflexes and rigidity were less marked, sensation had returned down to the groins, and the cystitis and incontinence had disappeared. There were two large bed-sores. A second operation was performed to remove the pressure on the cord which still remained. A large mass of the tumour was found to have grown backwards through the gap made in the spinal canal at the first operation. The first, fifth, and sixth dorsal laminæ were removed, but still no limit could be seen to the growth. The patient died eight hours later. Hale White remarked that dermoid cysts had been recorded as occurring in the middle line between the skin and spines, but not within the spinal canal.

**Treatment of Tic Douloureux.**—Joseph M. Spellissy[7] describes a case in which he removed the external two-thirds of the Gasserian ganglion by the Hartley-Krause method, after preliminary ligation of the external carotid artery. The writer remarks that it is too early yet by several years to announce that the neuralgia had been cured, but the case is published to add to the testimony concerning the utility or fatuity of ligating the external carotid in the operation. The patient was a blacksmith, aged 55 years. Right-sided facial neuralgia began nine years previously, following a sudden chilling after hard working. Several teeth were removed without relief. Four years ago the attacks of pain had become so severe and frequent that T. G. Morton had excised the right infra-orbital nerve. Eight months' relief of pain followed. Nine months later Morton removed the right inferior dental nerve. Eighteen months' relief of pain resulted. For the two years preceding the date of operation by Spellissy the painful attacks had increased in number, duration, and severity. Paroxysms of agonising pain were provoked by swallowing or by washing the face. Spellissy had a dread of hæmorrhage from the middle meningeal artery from what he had read and seen, and therefore decided to ligature the external carotid. Fowler had done this successfully in his second Gasserian ganglion operation. Fowler had adopted the plan in his third operation also, but had been so embarrassed by hæmorrhage on raising the dura from the base of the skull that he had to pack with gauze and attempt the completion of the operation later, when he again met with hæmorrhage which prevented removal of the ganglion. The favourable results L. McL. Tiffany obtained by excising the trunks of the second and third branches of the fifth nerve, and of the external two-thirds of the ganglion, *i.e.*, the part in continuity with those two trunks, led Spellissy to adopt that conservative plan of operating. From reported results the procedure seemed sufficiently radical, and free from the danger of loss of the eye which has followed the complete removal of the ganglion, with division of its ophthalmic branch. Post-mortem practice showed Spellissy the necessity, in cutting the bone flaps, of carrying the cuts down to the level of the upper border of the zygoma to ensure the flap hinging on a level with the floor of the skull. At the operation, though the middle meningeal artery tunnelled the bone of the flap and was cut transversely, there was no hæmorrhage. The dura was stripped up and then slightly slit to get rid of the cerebro-spinal fluid. An excellent view was then obtained. The two nerves were then divided by a cutting-hook, and the distal ends tucked into the foramina. The external portion of the ganglion was "dissected, cut, torn, and curetted away." Next day the previously hyperæsthetic areas of the face were found to be anæsthetic. Convalescence was uneventful. The eye was kept closed, and was washed several times a day with boric acid solution. Goggles were worn several weeks as a protection. During the two and a half months following the operation there was only one momentary twinge of pain. There was unequal action of the lower jaw, and a dribbling of saliva. It was intended at the operation to isolate and preserve the motor fibres of the third branch of the fifth, as recommended by Tiffany, but there had not been time. Professors W. W. Keen and William G. Spiller[8] report cases of resection of the fifth nerve, with an account of the microscopic appearances of the portions of nerve removed. Case I. was of a woman of 53, who had suffered in 1894 from pain, first at the inner angle of the right eye, and subsequently spreading to and involving the entire right face and anterior part of the scalp. From 1896 to 1898 she was free from the pain. In February, 1898, it returned suddenly and persisted. There was twitching of muscles about the right eye with the pain. The supra- and infra-orbital nerves were removed. She improved after the operation, and had no pain for 11 months, then a slight return, but with eventual disappearance. Spiller's examination of the nerves showed degeneration of the medullary sheaths in the form of minute balls stained black by osmic acid. Sections stained with carmine and Delafield's hæmatoxylin showed more or less round-celled infiltration around the small blood vessels. Many axis-cylinders appeared swollen and degenerating. Abbe[9] gives his experience of the surgery of the fifth nerve for tic with 20 cases, the Hartley operation having been done five times, the Salzer four, and the Carnochan 11. He advocates the last, with clean resection of the second branch to the foramen rotundum, for most bad cases, even where the first and third branches seem to share the neuralgic shocks. If the Salzer method is chosen he advises a section of the zygoma and turning it down with the skin, and muscle-splitting of the temporal rather than coronoid section. If the intra-cranial method is used he advocates simple section and excision of the second and third branches from the ganglion to their foramina, and the interposition of a piece of sterile rubber-tissue, and does not believe resection of the ganglion is necessary.

[5] Brit. Med. Jour., Feb. [6] Lancet, Mar. [7] Annals of Surgery, April. [8] Vide Lancet, May. [9] Jour. Amer. Med. Assoc., May, 1900.

### GENITO-URINARY SURGERY.

(*Continued from page* 355.)

**Wandering Kidney.**—Individual surgeons differ as to the estimation of a wandering or movable kidney, for to the normal kidney there is a natural anatomical respiratory movement of from two to five centimetres. The failure of the kidney to ascend after a forced inspiration is regarded as an adequate test by Mansell Moullin,[6] even if the kidney only comes low enough to enable its lower end to be felt. The term "wandering kidney" is, therefore, one which is relative to the movements of the diaphragm. Moullin has examined the anatomical and theoretical views of the subject in literature, and gives the following details:—

The kidneys are enclosed in the peri-renal fascia—a specialised portion of the sub-peritoneal tissue—supplemented on the left side by certain ventral fibrous bands (Zuckerkandl). This fascia comprises anterior and posterior layers, blended externally, but separate to the inner side and below. The kidney normally moves freely within this capsule, inside which is a special fatty capsule of a soft, delicate structure, immediately investing the kidneys. The capsule of the kidney is not the sole factor in the retention of the organ, for if the abdomen be opened with the body in the erect position the kidneys fall, as if retained *in situ* by the abdominal pressure. The suggestion is thrown out that the frequency of movable kidney on the right side may be due to a greater shallowness of the lumbar region there, owing to rotation of the transverse processes of the vertebræ in association with the lateral deviation of the lumbar vertebræ to the left, compensatory to the deviation to the right in the dorsal region of right-handed individuals. The origin of movable kidney cannot be said to be definitely known; but if this view were correct, we should expect the left kidney to be more liable to dislocation in left-handed persons—a supposition which does not seem probable.

A new method for fixing a movable kidney has been suggested by L. L. Arthur,[7] which consists in making a transverse incision below the last rib, and stripping up the peritoneum to the middle line posteriorly. A pocket is then made in the lumbar aponeurosis behind, and the lower end of the kidney is placed in the pouch so made. It is doubtful whether the kidney is a suitable organ to place in such a rigid pouch, so that the method is probably unnecessarily severe in view of the adequate operations of a simpler character.

**Syphilis.**—Some interesting facts in the treatment of this disease, according to present-day notions, have been given by that authority on the subject—Mr. Jonathan Hutchinson. Mercury is the established antidote to the disease, but is not essential to the treatment, for, like other specific fevers, syphilis may run its course and die out without treatment. Mercury, if given early, may prevent or greatly lessen secondary symptoms. As a cure the drug must be given over a long time to combat the low organisms of the disease.[8] Mr. Hutchinson is in favour of the removal of a chancre, if seen very early—within a week or so of contagion; but, of course, there are practical difficulties in the way. Then as to when mercury should be given, Mr. Hutchinson, unlike some other authorities, approves of its early administration, the earlier the better. He approves of giving mercury for a year, discontinuing it for a while, and then repeating it again. It does not matter much how the drug is given. Tonics may be given at the same time. In tertiary syphilis mercury and iodide are best administered separately.

Jonathan Hutchinson, jun., has given two very interesting lectures on syphilis,[9] pointing out the features of the early lesions, and the sites of various extra-genital chancres. He regards the bubo as being an almost constant accompaniment, with more constant features than the primary sore, and the presence of indurated lymphatics can be determined in half the cases. Concealed chancres in women are most common on the cervix uteri. He explains the greater severity of the late lesions of congenital syphilis by the fact that probably for months the presence of an initial chancre was overlooked, so that the virus obtained a firm hold. The presence of blood in the foul discharge from a chancrous foreskin should suggest phagedena. The following are the chief points in favour of the infective character of a sore: Solitary, or at most not several sores; length of time which elapses between the exposure and the appearance of the lesion; the constancy of a "bullety" bubo; induration; hard lymphatic cords. Tertiary ulceration of the penis is very apt to simulate a primary sore and so resist treatment. Reinduration at the site of a former hard chancre is an interesting circumstance during tertiary syphilis, unaccompanied by marked enlargement of the inguinal glands.

[6] Brit. Med. Jour., 1900, I., 566. [7] Boston Med. and Surg. Jour., cxli. [8] Brit. Med. Jour., 1900, I., 843. [9] Lancet, 1900, I., 1,575 and 1,637.

